# Association of peripheral neutrophil count with intracranial atherosclerotic stenosis

**DOI:** 10.1186/s12883-020-01641-3

**Published:** 2020-02-22

**Authors:** Xing Zhang, Xiao-He Hou, Ya-Hui Ma, Xue-Ning Shen, Xi-Peng Cao, Jing-Hui Song, Lan Tan, Jin-Tai Yu

**Affiliations:** 1 Department of Neurology, Qingdao Municipal Hospital, Dalian Medical University, Dalian, China; 2 Department of Neurology, Qingdao Municipal Hospital, Qingdao University, Qingdao, 266071 China; 3 Department of Neurology and Institute of Neurology, Huashan Hospital, Shanghai Medical College, Fudan University, 12th Wulumuqi Zhong Road, Shanghai, 200040 China; 4 Clinical Research Center, Qingdao Municipal Hospital, Qingdao University, Qingdao, China; 5 Department of Neurology, The Affiliated Hospital of Qingdao University, Qingdao, China

**Keywords:** Neutrophil, Inflammation, Atherosclerosis, Stroke, Magnetic resonance angiography

## Abstract

**Background:**

Inflammation plays an important role in atherosclerosis but the contribution of neutrophils to this process is unclear. We sought to assess whether neutrophil count is associated with intracranial atherosclerotic stenosis (ICAS).

**Methods:**

A total of 2847 individuals were included in our study, including 1363 with acute ischemic stroke and 1484 normal controls without stroke. The presence of ICAS was confirmed by magnetic resonance angiography. The association between neutrophil count and ICAS was evaluated by multivariable logistic regression analysis.

**Results:**

Among 2847 individuals included in this study, individuals with ICAS had higher neutrophil counts than those without ICAS in groups with and without stroke (*P* <  0.0001 for stroke group, *P* = 0.0097 for group without stroke). The multivariable logistic regression analysis showed that the third and fourth quartiles were independent predictors of ICAS in all the subjects (Q3: OR 1.81, 95% CI 1.39–2.37, Q4: OR 2.29, 95% CI 1.70–3.10) and patients in the fourth quartile had a higher risk for the occurrence of ICAS in stroke group (Q4: OR 2.82, 95% CI 1.79–4.48). However, there was no significant association between neutrophil count and ICAS in the group without stroke.

**Conclusions:**

The levels of circulating neutrophils were associated with the presence of ICAS. Our findings suggest that neutrophils may play a role in the pathogenesis of stroke related to ICAS and emphasize the need to develop proper strategies to control neutrophil response for the treatment of ICAS.

## Background

Stroke has attracted clinical and public health attention from all over the world. The number of patients with stroke, stroke-related deaths, physical disability, hospitalizations and the global burden are great and increasing [1, 2]. Accumulating evidence have shown that intracranial atherosclerotic stenosis (ICAS) is a major risk factor for ischemic stroke in Asia, especially in China, and is also known as an important pathogenetic mechanism of ischemic stroke [3–5]. At present, many researchers are paying more and more attention to the etiology of intracranial atherosclerosis. There is evidence suggesting that inflammatory processes contribute to both the initiation and progression of atherosclerosis [6, 7]. And even more importantly, an elevated number of activated of inflammatory markers have extremely important effect on the formation, evolution and destruction of atherosclerotic plaques [8, 9]. Atherosclerotic plaques were markedly infiltrated by various inflammatory cells, including activated macrophages, T cells, and mast cells [10–13]. These cells may provoke atherosclerotic plaque destabilization and the occurrence of acute thrombotic events by releasing matrix-degrading enzymes and thrombogenic substances [14–16]. Several studies have shown the correlation of increased peripheral inflammatory marker levels with various vascular diseases as well as the potential development and severity of atherosclerosis [6, 17], providing an indication that atherosclerosis is an inflammatory disease [18, 19]. Although intracranial atherosclerosis is viewed as a part of atherosclerotic disease, it is still unclear whether ICAS is also related to the inflammatory response.

Experimental studies on stroke have indicated that increased leukocytes enter into ischemic territory through chemotaxis of activated microglial cells within minutes of acute infarction onset [20]. Neutrophils are the first leukocyte subtype to infiltrate the areas of brain ischemia after cerebral infarctions and are considered to be markers of inflammation [21]. The elevated level of neutrophils accelerates inflammatory process by releasing various destructive materials (such as cytotoxins, reactive oxygen radicals, arachidonic acid, and proteolytic enzymes), which can cause damage to vascular endothelial cells and further promote the occurrence and progression of atherosclerosis [18, 22, 23]. Thus, endothelial dysfunction would might be an important pathophysiological link between neutrophils and ICAS. Several studies have suggested that elevation in neutrophil count is correlated with greater risk of ischemic brain injuries, vascular events as well as worsening functional outcomes [24–27]. However, the scientific information regarding the correlation of circulating neutrophils and ICAS is still lacking. Further studies to clarify the association are warranted.

Accordingly, the purpose of this study was to assess the relationship between peripheral neutrophil count and the presence of ICAS. The effect of neutrophil count on different locations of ICAS and ICAS burdens were also evaluated to help us develop a deeper understanding of ICAS.

## Methods

### Study population

Study participants were prospectively recruited from January 2014 to June 2018 among individuals in the Department of Neurology of Qingdao Municipal Hospital for suspected stroke and individuals underwent comprehensive physical examination at Medical Examination Center of Qingdao Municipal Hospital. Most of the subjects from medical examination center had no serious health problems. And the costs of physical exam were to be paid by their company or by each participants. We used the Trial of Org 10,172 in acute stroke treatment (TOAST) typing to classify stroke mechanism and NIHSS scoring system to assess the severity of acute stroke [28]. We excluded subjects who: (1) under 40 years old; (2) underwent incomplete angiography and laboratory examination; (3) had atrial fibrillation, cardiac embolism, vascular disease and had underwent replacement; (4) had intracranial and external artery dissection, arteritis, moyamoya disease, muscular fiber dysplasia; (5) had infection, nausea, tumor, chronic liver disease and renal insufficiency. Ethics approval for this study was obtained from the Institutional Ethics Committees of Qingdao Municipal Hospital.

Finally, 1484 subjects without stroke controls and 1363 stroke patients (without ICAS *n* = 2090, with ICAS *n* = 757) were included in this study. We have strict age and gender matching between the case and control subjects.

### Clinical and laboratory data

The baseline information were collected, including demographics, clinical characteristics, hypertension (defined as systolic ≥140 mmHg or diastolic ≥90 mmHg by repeating blood pressure measurement or having been treated with antihypertensive medication), diabetes mellitus (defined as fasting plasma glucose value ≥126 mg/dL, or 2-h postprandial plasma glucose ≥200 mg/dL, or glycosylated hemoglobin levels ≥6.5% for diabetes in multiple measurements, or having been treated with hypoglycemic medication), hyperlipidemia (defined as fasting total cholesterol level (TC) > 200 mg/dL or low-density lipoprotein cholesterol (LDL-C) ≥ 130 mg/dL or having received cholesterol-lowering drug), history of coronary heart disease, history of atrial fibrillation, history of stroke, smoking (current smoker or a patient who had quit smoking < 6 months previously) and alcohol use (defined as alcohol use > 2 units per day on average for men or > 1 unit per day on average for women).

All subjects underwent standardized laboratory examination. The fasting venous blood samples were obtained for biochemical analysis after 12 h of overnight fasting. Samples obtained through venipuncture were collected into test tubes containing anticoagulated blood. The neutrophil counts were tested using automated blood cell counter at the hematology laboratory on admission.

### Imaging collection and analysis

All subjects were imaged with 1.5-Tesla or 3.0-Tesla MR scanners, including the sequence of diffusion weighted image (DWI), magnetic resonance imaging(MRI) and three-dimensional time-of-flight magnetic resonance angiography(MRA). ICAS was defined as the presence of 50–99% stenosis or the occlusion of the intracranial vessels according to Warfarin-Aspirin Symptomatic Intracranial Disease (WASID) trial criteria [29, 30]. To assess the correlation between neutrophil count and the number of ICAS, we counted the number of vascular lesions as single, two or multiple (more than three). To analyse the effect of neutrophil count on different vasular stenosis sites,we dichotomized the locations of ICAS into anterior circulation, posterior circulation and both anterior circulation and posterior circulation. Two experienced neurologists, who were blinded to the participants’ clinical characteristics and biochemical results, visually reviewed all magnetic angiographic images to assess the presence and degree of ICAS lesions.

### Statistical analysis

Results were expressed as mean ± standard deviation (SD) for continuous variables with normal distributions, and as median with interquartile range for the continuous skewed distributed data. Categorical data were presented as frequencies and proportions. The Shapiro-Wilk test was performed to evaluate the normality for the distribution of continuous variables. The baseline variables were compared between groups using Mann-Whitney U-test. Kruskal-Wallis test is used if the comparison is conducted among more than two groups. Differences in categorical variables were compared using a χ^2^ test. Neutrophil count was divided into quartiles. Logistic regression analysis was performed across neutrophil quartiles to examine the association of neutrophil count with ICAS. Differences in associations of neutrophil count with ICAS presence or absence were tested for statistical significance in a multivariable analysis. Multivariable regression was used to correct for possible confounding variables, which included those potential determinants of outcome that had significant clinical correlation or showed *P* values < 0.05 in univariate analysis.

We compared neutrophil count among single, two and multiple ICAS groups using the Kruskal-Wallis test. To analyse the effect of neutrophil count on different vasular stenosis sites, we compared neutrophils among subgroups according to the vascular circulations. We classified the location of anterior and posterior circulation according to the classification method mentioned in the current literature [31]. *P* <  0.05 was considered to indicate a significant difference. Statistical analyses were performed using the R statistical software (version 3.4.3).

## Results

### Baseline characteristics

A total of 2847 individuals were included in this study, including 1363 with acute ischemic stroke (AIS) and 1484 without stroke controls. All the participants were further categorized into four groups: in stroke group, there are 474 (34.78%) patients with ICAS and 889 (65.22%) without ICAS; in the group without stroke, there are 283 (19.07%) subjects with ICAS and 1201 (80.93%) without ICAS.

The baseline characteristics of the study population are summarized in Table 1. The mean age of these subjects was 68.43 ± 11.17 (range, 40–97) years, and 1739 (61.08%) individuals were male. The levels of circulating neutrophils in stroke group were higher than those in the group without stroke (*P* <  0.0001). Compared to subjects without stroke, these patients with acute cerebral infarction had a higher level of neutrophils, a higher neutrophil-to-lymphocyte ratio and a lower level of lymphocytes. We further tried to look into the difference in neutrophil levels between males and females. We found that men had higher levels of neutrophils than women (*P* <  0.0001).

**Table 1:** Baseline Characteristics of the study population in the present study

	With stroke(*n* = 1363)	Without stroke(*n* = 1484)	*P*-value
Characteristics			
Age, mean ± SD, y	68.44 ± 11.65	68.41 ± 10.70	0.7669
Male, *n* (%)	835(61.26%)	904 (60.92%)	0.8502
Hematological examinations			
Lipoprotein, median (IQR), mg/dL	16.120 (7.270–30.320)	13.315 (6.065–25.902)	< 0.0001
Triglyceride, median (IQR), mmol/L	1.320 (0.990–1.790)	1.220 (0.890–1.760)	< 0.0001
Total cholesterol, median (IQR), mmol/L	5.030 (4.210–5.885)	4.725 (4.000–5.480)	< 0.0001
HDL, median (IQR), mmol/L	1.110 (0.950–1.300)	1.110 (0.940–1.330)	0.4013
LDL, median (IQR), mmol/L	3.110 (2.550–3.700)	2.860 (2.320–3.413)	< 0.0001
Neutrophils, median (IQR), (per 10^9^/L)	4.100 (3.230–5.400)	3.470 (2.770–4.322)	< 0.0001
Lymphocytes, median (IQR), (per 10^9^/L)	1.900 (1.490–2.410)	1.960 (1.540–2.450)	0.0986
NLR, median (IQR)	2.060 (1.540–3.080)	1.700 (1.300–2.373)	< 0.0001
Glucose, median (IQR),mmol/L	5.520 (4.630–7.445)	5.270 (4.697–6.433)	0.0001
Medical history, *n* (%)			
Hypertension, *n* (%),	1036 (76.01%)	1094 (73.72%)	0.1598
Diabetes mellitus, *n* (%)	505 (37.05%)	440 (29.65%)	< 0.0001
Coronary Heart Disease, *n* (%)	434 (31.84%)	607 (40.90%)	< 0.0001
Smoking, *n* (%)	479 (35.14%)	432 (29.11%)	0.0006
Alcohol use, *n* (%)	345 (25.31%)	269 (18.13%)	< 0.0001
Stenosis, *n* (%)	474 (34.78%)	283 (19.07%)	< 0.0001

Abbreviations: *IQR* interquartile range, *NLR* neutrophil to lymphocyte ratio, *SD* standard deviation, *HDL* high-density lipoprotein, *LDL* low-density lipoprotein

### Association between neutrophil count and ICAS

The levels of circulating neutrophils in subjects with ICAS were higher than those in subjects without ICAS (P <  0.0001).

**Fig. 1:**
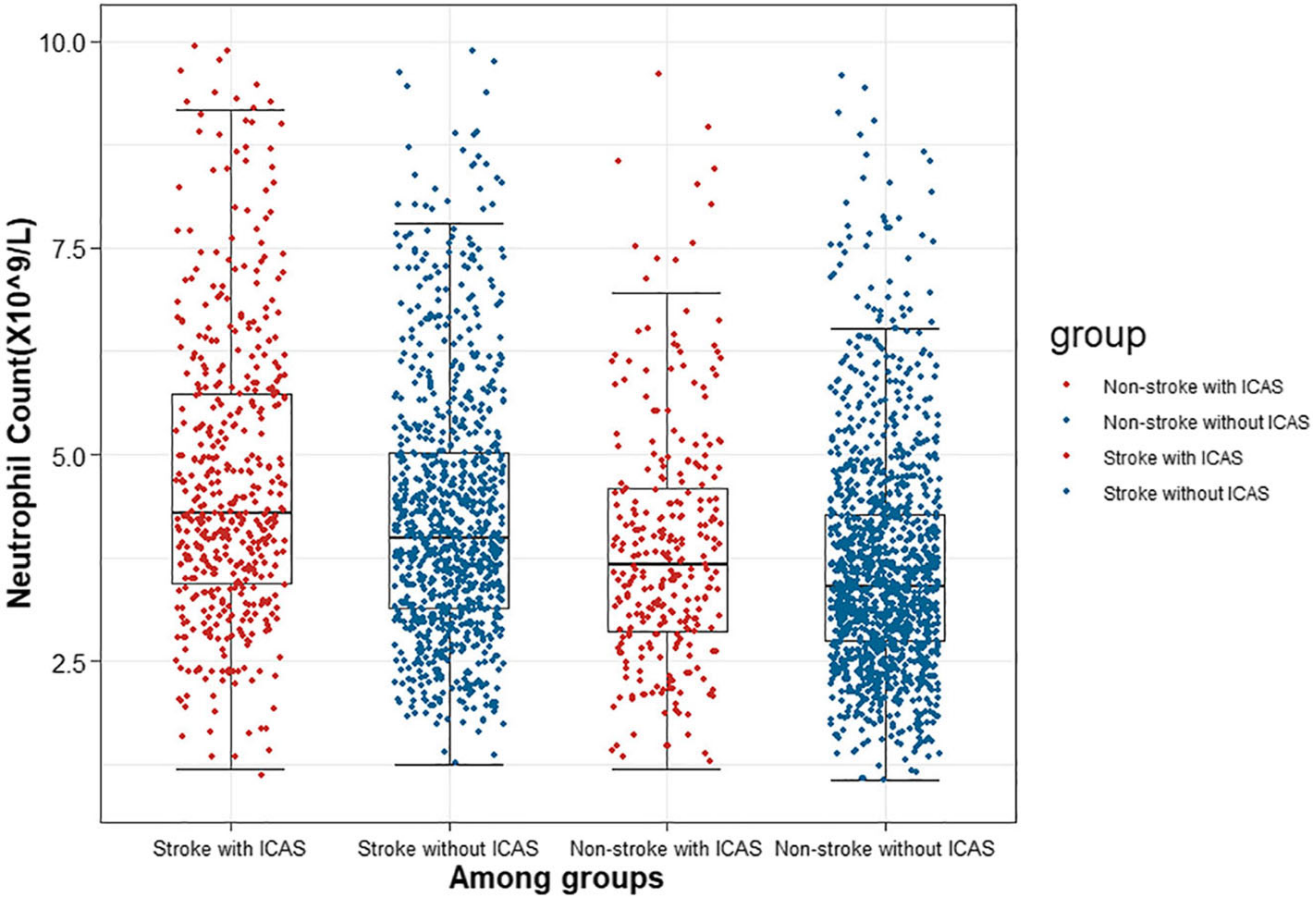
The levels of neutrophil count among four groups of participants. *The neutrophil counts were significantly higher in subjects with ICAS than those of subjects without ICAS in groups with and without stroke (*P* <  0.0001 for stroke group, *P* = 0.0097 for group without stroke)

The multivariable logistic regression analysis showed that the third and fourth quartiles were independent predictors of ICAS in all the subjects (Q3: OR 1.81, 95% CI 1.39–2.37, Q4: OR 2.29, 95% CI 1.70–3.10) and patients in the fourth quartile had a higher risk for the occurrence of ICAS in stroke group (Q4: OR 2.82, 95% CI 1.79–4.48) (Table 3). To evaluate the correlation between peripheral neutrophil count and ICAS lesions, we further carried out a subgroup analysis. Baseline characteristics of these subjects with and without ICAS are shown in Table 2. In subgroup analysis, the neutrophil counts were significantly higher in subjects with ICAS than those of subjects without ICAS in groups with and without stroke (*P* <  0.0001 for stroke group, *P* = 0.0097 for group without stroke) (Fig. 1). The multivariable logistic regression analysis showed that each quartile was associated with the occurrence of ICAS in stroke group (Q2: OR 1.44, 95% CI 1.00–2.08, Q3: OR 1.89, 95% CI 1.26–2.86, Q4: OR 2.82, 95% CI 1.79–4.48), whereas the association in the group without stroke was not significant (Table 3).

**Table 2:** Characteristics of the study population in the present study according to stroke and ICAS status

	With stroke(*n* = 1363)		P-value	Without stroke(*n* = 1484)		*P*-value
	With ICAS(*n* = 474)	Without ICAS(*n* = 889)		With ICAS(*n* = 283)	Without ICAS(*n* = 1201)	
Characteristics						
Age, mean ± SD, y	69.15 ± 11.85813	68.07 ± 11.53326	0.0665	69.3 ± 11.17	68.2 ± 10.58	0.0667
Male, *n* (%)	291 (61.39%)	544 (61.19%)	0.9424	170 (60.07%)	734 (61.12%)	0.7458
Hematological examinations						
Lipoprotein, median (IQR), mg/dL	17.14 (7.36–31.66)	15.84 (7.20–28.83)	0.2408	15.250 (6.605–27.635)	13.02 (5.92–25.15)	0.0977
Triglyceride, median (IQR), mmol/L	1.3000 (0.9925–1.7300)	1.320 (0.980–1.850)	0.6036	1.230 (0.930–1.785)	1.210 (0.880–1.750)	0.7941
Total cholesterol, median (IQR), mmol/L	5.020 (4.162–5.800)	5.040 (4.240–5.940)	0.3962	4.510 (3.710–5.395)	4.770 (4.070–5.490)	0.0064
HDL, median (IQR), mmol/L	1.055 (0.910–1.280)	1.130 (0.970–1.320)	0.0010	1.060 (0.885–1.280)	1.130 (0.960–1.340)	0.0002
LDL, median (IQR), mmol/L	3.105 (2.493–3.610)	3.120 (2.580–3.730)	0.3264	2.800 (2.070–3.425)	2.870 (2.360–3.410)	0.0364
Neutrophils, median (IQR), (per 10^9^/L)	4.320 (3.493–5.880)	3.990 (3.150–5.040)	< 0.0001	3.670 (2.850–4.625)	3.420 (2.750–4.270)	0.0097
Lymphocytes, median (IQR), (per 10^9^/L)	1.835 (1.433–2.348)	1.950 (1.510–2.430)	0.0288	1.810 (1.425–2.385)	1.990 (1.580–2.470)	0.0018
NLR, median (IQR)	2.270 (1.712–3.522)	1.970 (1.490–2.840)	< 0.0001	1.830 (1.345–2.590)	1.680 (1.280–2.330)	0.0025
Glucose,median(IQR), mmol/L	5.845 (4.770–7.670)	5.390 (4.580–7.290)	0.0067	5.810 (4.850–8.500)	5.200 (4.660–6.120)	< 0.0001
Medical history, *n* (%)						
Hypertension, *n* (%),	381 (80.38%)	655 (73.68%)	0.0058	232 (82.27%)	862 (58.09%)	0.0005
Diabetes mellitus, *n* (%)	213 (44.97%)	292 (32.85%)	< 0.0001	100 (35.34%)	340 (28.31%)	0.0199
Coronary Heart Disease, *n* (%)	164 (34.60%)	270 (30.37%)	0.1105	110 (38.87%)	497 (41.38%)	0.4392
Smoking, *n* (%)	152 (32.07%)	327 (36.78%)	0.0825	100 (35.34%)	332 (22.37%)	0.0104
Alcohol use, *n* (%)	108 (22.78%)	237 (26.66%)	0.1172	66 (23.32%)	203 (13.68%)	0.0117

Abbreviations: *IQR* interquartile range, *ICAS* intracranial atherosclerotic stenosis, *NLR* neutrophil to lymphocyte ratio, *SD* standard deviation, *HDL* high-density lipoprotein, *LDL* low-density lipoprotein

**Table 3:** Logistic regression analysis of the association between neutrophil count and intracranial atherosclerotic stenosis according to quartiles of neutrophil count

	Unadjusted			Multivariate Analysis		
	OR	95%CI	*P*-value	OR	95%CI	*P*-value
With stroke						
Neutrophil(total)	1.18	1.11–1.25	< 0.0001	1.22	1.12–1.34	< 0.0001
Quartile 1 (< 3.23)	ref			ref		
Quartile 2 (3.23–4.09)	1.18	0.85–1.64	0.3310	1.44	1.00–2.08	0.0499
Quartile 3 (4.10–5.40)	1.27	0.91–1.75	0.1560	1.89	1.26–2.86	0.0024
Quartile 4 (> 5.40)	2.17	1.58–2.99	< 0.0001	2.82	1.79–4.48	< 0.0001
Without stroke						
Neutrophil(total)	1.10	1.01–1.19	0.0205	1.19	1.03–1.38	0.0214
Quartile 1 (< 2.77)	ref			ref		
Quartile 2 (2.77–3.46)	0.94	0.64–1.39	0.7632	0.91	0.60–1.39	0.6731
Quartile 3 (3.47–4.32)	1.37	0.95–1.98	0.0910	1.39	0.92–2.11	0.1228
Quartile 4 (> 4.32)	1.39	0.96–2.01	0.0807	1.47	0.89–2.43	0.1302
Total						
Neutrophil(total)	1.19	1.14–1.25	< 0.0001	1.24	1.16–1.32	< 0.0001
Quartile 1 (< 2.94)	ref			ref		
Quartile 2 (2.94–3.74)	1.04	0.81–1.35	0.7390	1.02	0.78–1.34	0.8924
Quartile 3 (3.75–4.80)	1.63	1.28–2.08	< 0.0001	1.81	1.39–2.37	< 0.0001
Quartile 4 (> 4.80)	2.09	1.65–2.66	< 0.0001	2.29	1.70–3.10	< 0.0001

Abbreviations: *OR* Odd Ratio, *CI* Confidence Interval, *ICAS* intracranial atherosclerotic stenosis. In the group with stroke: adjusted for high-density lipoprotein, Lymphocytes, neutrophil to lymphocyte ratio, hypertension, diabetes mellitus, glycemia levels. In the group without stroke: adjusted for total cholesterol, high-density lipoprotein, low-density lipoprotein, lymphocytes, neutrophil to lymphocyte ratio, hypertension, diabetes mellitus, smoking, alcohol use, glycemia levels. Total: adjusted for lipoprotein, triglyceride, total cholesterol, high-density lipoprotein, low-density lipoprotein, neutrophil to lymphocyte ratio, diabetes mellitus, smoking, alcohol use, coronary heart disease, glycemia levels. In the group with stroke: among stroke with ICAS and stroke without ICAS groups; In the group without stroke: among non-stroke with ICAS and non-stroke without ICAS groups; Total: among groups with and without stroke

### Association between neutrophil count and the number and locations of ICAS

**Fig. 2:**
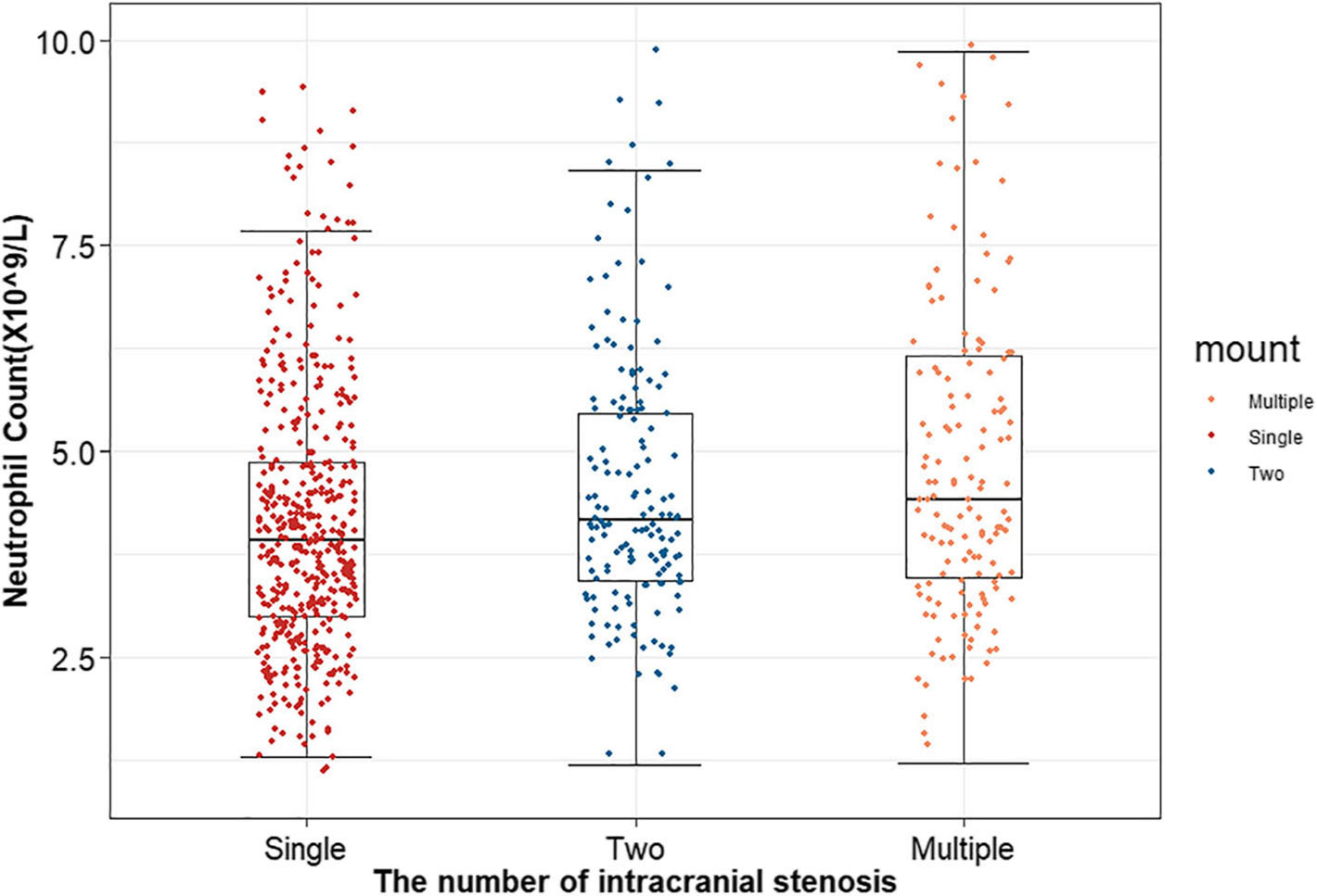
Differences in neutrophil count among groups according to the number of intracranial stenosis. *In multiple ICAS group, individuals had higher levels of neutrophil than those in other two groups (*P* <  0.0001)

**Fig. 3:**
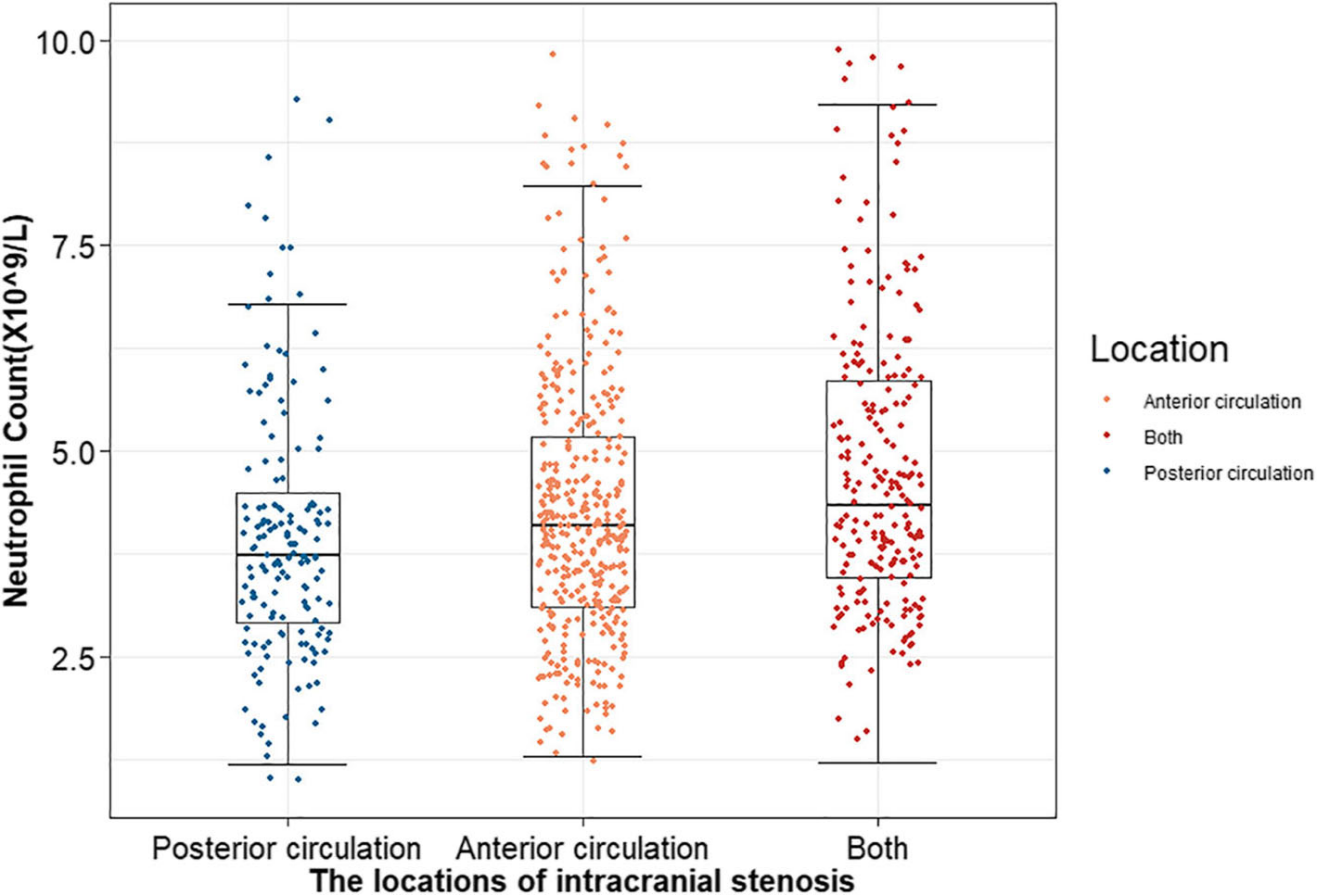
Differences in neutrophil count among groups according to ICAS lesion locations. *The highest count of neutrophils was found in the group with both anterior and posterior circulation ICAS (*P* <  0.0001)

Among these participants, 757 (26.59%) of them were found to have ICAS, including 445 patients with single ICAS, 159 patients with two ICAS, and 153 patients with multiple ICAS (more than three). We further evaluated the correlation between neutrophil count and the number of ICAS. We found that patients in multiple ICAS group had higher levels of neutrophils than those in the other two groups (*P* <  0.0001) (Fig. 2). In addition, the effect of neutrophil count on different locations of ICAS was also analysed, and the highest count of neutrophils was found in the group with both anterior and posterior circulation ICAS (P <  0.0001) (Fig. 3).

## Discussion

The major finding of this study was that peripheral neutrophil count was associated with ICAS. Higher neutrophil level was associated with greater risk of ICAS in patients with AIS, but there was no significant correlation in subjects without stroke. In addition, we also found that the number and locations of ICAS were associated with the neutrophil count. Neutrophils might play an important role in the presence of ICAS. Given the difference in neutrophil count between subjects with and without stroke, neutrophils may increase the occurrence of symptomatic intracranial atherosclerotic stenosis.

Many studies have indicated that vascular risk factors and metabolic syndrome have been considered to be crucial determinants of ICAS [5, 27, 32]. Atherosclerosis is increasingly considered to be a chronic inflammatory disease [18, 19], which plays a pivotal role in the development and destabilization of atherosclerosis [6, 7]. Neutrophils, as major determinants of inflammation, have become the focus in exploring the relationship between neutrophil levels and vascular stenosis. Previous studies confirmed that coronary artery stenoses are associated with high neutrophil count in patients with chronic stable angina [33]. Studies have also shown that carotid artery disease has a relationship with neutrophils in patients with peripheral arterial disease [34].

Stroke is closely related to intracranial atherosclerotic disease, which occurs through various mechanisms such as artery-to-artery embolism, hemodynamic insufficiency, situ thrombotic occlusion, and branch occlusion [5].In the Northern Manhattan Study, intracranial atherosclerosis caused ischemic stroke in 9% of white individuals, 17% of African-American, and 15% of Hispanic. Intracranial stenosis was reported in 33–37% of Chinese patients admitted to hospital with ischemic stroke [31]. Several studies indicated that AIS was associated with increased circulating markers of the inflammatory response, including peripheral neutrophil count, peripheral leukocyte count, C-reactive protein and so on [24, 35]. Similar to these studies, our study demonstrated that there existed high levels of neutrophils in patients with AIS. The increased number of activated peripheral neutrophils could result in atherosclerotic lesions by increased adhesion to and damage of the endothelial tissue as well as the secretion of destructive components [8]. Therefore, endothelium cell dysfunction would be an indispensable pathophysiological link between neutrophils and ICAS lesions.

The potential mechanism associating neutrophils with ICAS remains unclear, and the following mechanisms might explain their relationship. Firstly, neutrophils have been regarded as major components of the inflammatory response, which are able to generate detrimental materials when activated [22, 26]. These substances not only regulate acute inflammatory response, but also result in endothelial dysfunction [22, 36]. Research has suggested that endothelial impairment is deemed to be an early marker for atherosclerotic lesions and is beneficial to all stages of atherosclerosis [22, 36]. Given this relationship between endothelial dysfunction and atherosclerosis, there might be an important pathophysiological link between neutrophils and ICAS. Secondly, previous studies indicated that an elevated level of leukocytes was correlated with diabetes, obesity and hypertriglyceridemia, hence supporting the statement that inflammation is part of the metabolic syndrome [37, 38]. Studies have emphasized that metabolic syndrome was closely associated with the progress of atherosclerosis [32, 37]. These evidences imply that neutrophils may lead to atherosclerosis by affecting metabolic condition. Moreover, some studies have suggested that levels of inflammatory markers involved in the process of atherosclerosis are associated with vascular risk factors [39, 40]. Therefore, the mechanism underlying the association between neutrophils and ICAS may be that there are larger burdens of various vascular risk factors in subjects with increased neutrophil count. Further research is needed to identify possible mechanisms.

Interestingly, we found that the number of ICAS had correlation with circulating neutrophil count. Many studies have shown that the severity of coronary heart disease and carotid stenosis has association with inflammatory markers [6, 18, 27, 33, 34, 41]. We found that the neutrophil count was significantly higher in the multiple ICAS group, indicating that the greater number of ICAS may be associated with the higher levels of neutrophils. We also found that ICAS locations had correlation with elevated neutrophil count. However, a previous study demonstrated that there was no significant correlation between ICAS locations and inflammation [42], considering that atherosclerosis is a systemic disease. The result was inconsistent with our study. The inconsistency may be due to the heterogeneity of ethnicity, differences in arterial or genetic susceptibility, regional difference and study design of different studies. A prior study indicated that different locations of intracranial stenosis are associated with different vascular risk factors and demographic features [43], whereas the mechanisms associating neutrophil count with vascular stenosis locations are not well known. We need to explore the underlying mechanism between them in future research. We further assessed the difference in neutrophil levels between genders. Compared to women, men had higher levels of neutrophils. The result may indicate that men were more likely to have ICAS than women. This result is consistent with a published article [44].

This study had several limitations. First, since this is a cross-sectional study, definitive conclusions on the association between neutrophils and ICAS cannot be reached. Further prospective studies are warranted to confirm our findings. Second, this is a single center study. Although we had a relatively large sample size, selection biases and race difference should also be taken into account. Thus, the application of our results to different demographic groups should be approached cautiously. Third, our study found that there is no relationship between neutrophil count and ICAS in the group without stroke. This result may be due to that the sample size is not large enough to reflect the actual relationship between neutrophils and ICAS. Therefore, we need to expand the sample size to further explore the association between neutrophils and ICAS in the group without stroke. Fourth, we measured the neutrophil level at a single time point and the acute stage of stroke. The dynamic change of neutrophil counts at different stages could not be presented in this study.

## Conclusion

To summarize, neutrophil count was correlated with the presence of ICAS. We also found that the number and locations of ICAS had relationship with neutrophils. Our findings suggest that neutrophils may play a role in the pathogenesis of stroke related to ICAS and emphasize the need to develop proper strategies to control neutrophil response for the treatment of ICAS. More studies will be needed to better evaluate the correlation between peripheral neutrophil count and ICAS.

## Data Availability

The datasets are available from the corresponding author on reasonable request.

## Abbreviations

AIS: Acute ischemic stroke
CI: Confidence Interval
DWI: Diffusion weighted image
FLAIR: Fluid attenuated inversion recovery imaging
ICAS: Intracranial atherosclerotic stenosis
LDL-C: Low-density lipoprotein cholesterol
MRA: Magnetic resonance angiography
MRI: Magnetic resonance imaging
OR: Odd Ratio
Q3: Quartile 3
Q4: Quartile 4
SD: Standard deviation
TC: Total cholesterol level
TIA: Transient ischemic attacks
WASID: Warfarin-Aspirin Symptomatic Intracranial Disease
